# Serotonin receptor HTR4 as a counter actor of lipid-induced increases of serum glucagon-like peptide-1 levels

**DOI:** 10.1007/s00424-020-02454-6

**Published:** 2020-08-22

**Authors:** Andreas Breit, Thomas Gudermann

**Affiliations:** Walther Straub Institute of Pharmacology and Toxicology, Medical Faculty, LMU Munich, Goethestrasse 33, 80336 Munich, Germany

In this issue of *Pflügers Archiv—European Journal of Physiology*, Okumura et al. present a new association between the serotonin and the GLP-1 system which, in turn, impacts serum insulin levels. In detail, they show a link between HTR4 activity in GLP-1 and PYY positive murine ileal epithelial cells and plasma levels of GLP-1 in mice. They first performed immunofluorescence staining to analyze HTR expression in murine ileum and colon. They found significant expression of HTR2A, HTR2B, and HTR4 expression in epithelial cells of both tissues. Interestingly, in the ileum, HTR4 was found in enteroendocrine (EE) L cells, a group of EE cells in the small intestine, which are also positive for GLP-1 and PYY as well as for serotonin (5-HT) expression. To validate these data, the authors also isolated small intestinal crypts from mice and cultured the dissociated cells. Here they could confirm that HTR4 positive cells co-express GLP-1 and PYY. These observations lead to the reasonable assumption that HTR4 signaling might affect GLP-1 and/or PYY release from these enteroendocrine L cells. In order to determine a potential association between HTR4 signaling and GLP-1 release, the authors monitored plasma GLP-1 levels from mice treated either intraperitoneally or intragastrically with the established selective HTR4 agonist tegaserod. Under these “basal conditions,” no effects of the HTR4 agonist on serum GLP-1 levels were detectable. However, when lipid-induced increase of serum GLP-1 levels was mimicked by administration of so-called Intralipos, a soybean oil emulsion, intragastric administration of tegasteron 30 min before significantly reduced Intralipos-induced increases of serum GLP-1 levels. This effect was inhibited by the co-administration of the HTR4 antagonist RS39604 confirming the importance of HTR4 signaling for this process. Interestingly, intraperitoneal administration of tegasteron failed to block Intralipos-induced increases of serum GLP-1 levels.

GLP-1 together with the gastric inhibitory polypeptide (GIP) are the two principle incretin hormones. Incretins are metabolic hormones that are released after eating and decreased blood glucose by augmentation of insulin secretion from pancreatic beta cells of the islets of Langerhans. Incretins are responsible for the observation that intravenous injection of glucose leads to less insulin secretion compared with oral glucose administration: the so-called incretin effect. Given the strong interaction between the GLP-1 system and insulin/glucose serum levels, it is not surprising that the GLP-1 system is involved in pathological states such as type 2 diabetes, obesity, and non-alcoholic fatty liver disease (NAFLD) [1–4]. Hence, synthetic GLP-1 receptor agonists such as liraglutide (Victoza) have been approved for medical use in Europe in 2009 and in the USA in 2010.

The new association between the serotonin and the GLP-1 system which impacts serum insulin levels as shown here opens up a new avenue to positively manipulate the GLP-1 system and as a secondary effect serum insulin levels under pathological conditions. The most intriguing part of this new strategy is the observation that HTR4 agonists affect serum GLP-1 levels only when increased by lipids and when applied intragastrically. This kind of specificity is responsible for the fact that the most wanted effects occur exactly at the desired time point: shortly after food uptake. Thus, it will be an enlightening and important task for upcoming studies to firstly reveal whether HTR4 signaling affects serum GLP-1 and thus insulin levels in humans and, secondly, whether these effects are beneficial for patients with type 2 diabetes.
